# Deletion of the chloroplast LTD protein impedes LHCI import and PSI–LHCI assembly in *Chlamydomonas reinhardtii*

**DOI:** 10.1093/jxb/erx457

**Published:** 2017-12-30

**Authors:** Jooyeon Jeong, Kwangryul Baek, Jihyeon Yu, Henning Kirst, Nico Betterle, Woongghi Shin, Sangsu Bae, Anastasios Melis, EonSeon Jin

**Affiliations:** 1 Department of Life Science and Research Institute for Natural Sciences, Hanyang University, Seoul, Korea; 2 School of Biological Sciences, Seoul National University, Seoul, Korea; 3 Department of Plant and Microbial Biology, University of California, Berkeley, California USA; 4 Department of Biology, Chungnam National University, Daejeon, Korea; 5 Department of Chemistry, Hanyang University, Seoul, Korea

**Keywords:** *Chlamydomonas reinhardtii*, chloroplast signal recognition particle pathway, CRISPR–Cas9, LHCP translocation defect (LTD), light-harvesting chlorophyll- and carotenoid-binding proteins, photosystem I

## Abstract

Nuclear-encoded light-harvesting chlorophyll- and carotenoid-binding proteins (LHCPs) are imported into the chloroplast and transported across the stroma to thylakoid membrane assembly sites by the chloroplast signal recognition particle (CpSRP) pathway. The LHCP translocation defect (LTD) protein is essential for the delivery of imported LHCPs to the CpSRP pathway in *Arabidopsis*. However, the function of the LTD protein in *Chlamydomonas reinhardtii* has not been investigated. Here, we generated a *C. reinhardtii ltd* (*Crltd*) knockout mutant by using CRISPR–Cas9, a new target-specific knockout technology. The *Crltd1* mutant showed a low chlorophyll content per cell with an unusual increase in appressed thylakoid membranes and enlarged cytosolic vacuoles. Profiling of thylakoid membrane proteins in the *Crltd1* mutant showed a more severe reduction in the levels of photosystem I (PSI) core proteins and absence of functional LHCI compared with those of photosystem II, resulting in a much smaller PSI pool size and diminished chlorophyll antenna size. The lack of CrLTD did not prevent photoautotrophic growth of the cells. These results are substantially different from those for Arabidopsis *ltd* null mutant, indicating LTD function in LHCP delivery and PSI assembly may not be as stringent in *C. reinhardtii* as it is in higher plants.

## Teaser

The *Chlamydomonas reinhardtii* LTD null mutant generated by CRISPR–Cas9, displayed aberrant PSI–LHCI holocomplexes, suggesting that the LTD protein may selectively function in PSI–LHCI assembly in green microalgae

## Introduction

Photosynthesis relies on the linear coordinate function of two photosystems (PSI and PSII), which are light-absorbing complexes in the thylakoid membrane of photosynthetic organisms (Taiz and Zeiger, 2010). Light-harvesting chlorophyll- and carotenoid-binding proteins (LHCPs) contain the photosynthetic pigments and function to absorb the energy of sunlight and transfer it to the reaction centers of the photosystems. LHCPs are also responsible for dissipating excess energy that can be harmful to the photosynthetic apparatus (Wobbe *et al.*, 2016). LHCPs include LHCA and LHCB, which form the light-harvesting complex I (LHCI) associated with PSI and the light-harvesting complex II (LHCII) associated with PSII, respectively. Since the *LHC* gene family is encoded in the nuclear genome (Jansson, 1999; Stauber *et al.*, 2003), LHCPs are synthesized in the cytosol and are then imported into the chloroplast. Once an LHCP is inside the chloroplast, it is transported across the stroma to its final destination, the thylakoid membrane assembly sites, by the so-called chloroplast signal recognition particle (CpSRP) pathway (Henry, 2010; Richter *et al.*, 2010).

In the higher plant Arabidopsis, CpSRP pathway components such as CpSRP54 and CpSRP43 associate with imported LHCPs and form a transit complex to maintain solubility of the hydrophobic LHCPs (Li *et al.*, 1995; Schuenemann *et al.*, 1998; Tzvetkova-Chevolleau *et al.*, 2007). CpSRP54 binding to the LHCPs increases the affinity of CpSRP43 for LHCP binding (Liang *et al.*, 2016). When the transit CpSRP54–LHCP–CpSRP43 complex reaches the thylakoid membrane surface, CpSRP43 interacts with the C-terminal tail of the ALB3 translocase, causing the CpSRP43 to release the LHCP enabling insertion of the LHCP into the developing thylakoid membrane (Dünschede *et al.*, 2011; Horn *et al.*, 2015; Liang *et al.*, 2016). Recruitment of ALB3 requires the CpSRP receptor CpFTSY (Moore *et al.*, 2003; Asakura *et al.*, 2008). CpFTSY is activated by anionic phospholipids and forms a transient complex with CpSRP54, thereby facilitating the binding of the ALB3 to the CpSRP54–LHCP–CpFTSY complex (Chandrasekar and Shan, 2017).

In addition to the above LHCP transport proteins, the LHCP translocation defect (LTD) protein has been reported to function in the stroma of chloroplasts (Cui *et al.*, 2011; Ouyang *et al.*, 2011). Upon the LHCP release from the Tic-Toc chloroplast envelope translocon complex, LTD first binds to the third transmembrane domain of the LHCP and delivers it to CpSRP43. An Arabidopsis *ltd* null mutant showed a drastic lowering of total chlorophyll (Chl) and LHCP and no growth under photoautotrophic conditions. These severe phenotypes suggested a critical role of the LTD protein in LHCP trafficking (Cui *et al.*, 2011; Ouyang *et al.*, 2011).

The genes of the CpSRP pathway proteins are present not only in higher plants but also in various eukaryotic photosynthetic microorganisms, ranging from chlorophytes to heterokonts. In the green microalga *Chlamydomonas reinhardtii*, the homologs of most CpSRP components have been identified and null mutants of each CpSRP protein have been characterized (Bellafiore *et al.*, 2002; Ossenbühl *et al.*, 2004; Göhre *et al.*, 2006; Kirst *et al.*, 2012a, b; Jeong *et al.*, 2017). However, to the best of our knowledge, no LTD deletion mutants have been reported in green algae. Here, we generated an LTD null mutant in *C. reinhardtii* (*Crltd*) by using the CRISPR–Cas9 method, a new target-specific knockout technology that has recently been successfully applied to *C. reinhardtii* (Baek *et al.*, 2016). In the *Crltd1* mutant, total Chl content was decreased to 33% of the wild-type level, but the Chl *a*/*b* ratio was not changed. The level of the PSI-LHCI complex in the mutant was severely reduced, to a far greater extent than that of the PSII-LHCII complex. Concomitantly, the ultrastructure of the thylakoid membranes was drastically altered. These results suggest that the CrLTD is specifically involved in the transport and assembly of the PSI-LHCI complexes, and that the associated *Chlamydomonas* CpSRP pathway may act differently in microalgae than in higher plants.

## Materials and methods

### Cell growth conditions

*Chlamydomonas reinhardtii* wild-type strain CC-4349 *cw15 mt−* (Baek *et al.*, 2016; Jeong *et al.*, 2017) and CRISPR–Cas9-induced knockout mutants were cultivated mixotrophically in Tris–acetate–phosphate (TAP) medium, or photoautotrophically in Tris–bicarbonate–phosphate (TBP), or high-salt medium (Harris, 1989) with continuous air bubbling under continuous illumination (50 or 100 μmol photons m^−2^ s^−1^) at 25 °C. For the growth analysis of cells, cultures were photoautotrophically grown in 400 ml Tris–phosphate medium with light intensities ranging from 100 to 350 μmol photons m^−2^ s^−1^. A 500 ml laboratory glass bottle (Duran) was employed with continuous 3% CO_2_ bubbling to avoid carbon limitation. The cell growth rate (μ) was measured during the exponential growth phase from the increase in cell number as a function of time, following the method described in Levasseur *et al.* (1993).

### CRISPR–Cas9 driven mutagenesis

All procedures were performed according to Baek *et al.* (2016) and Yu *et al.* (2017). Recombinant Cas9 protein (200 μg) and *in vitro* transcribed single guide RNA (sgRNA; 140 μg) were mixed and preincubated for 10 min at room temperature. Then, *Chlamydomonas* cells (5 × 10^5^ cells) were transformed with the ribonucleoprotein (RNP) complex in a Gene Pulser Xcell Electroporation System (Bio-Rad). After transformation, cells were diluted and plated on TAP medium containing 1.5% agar to obtain single colonies for further investigation. For genotype characterization, genomic DNA was extracted as described in Jeong *et al.* (2017) and DNA segments were analysed as given by Baek *et al.* (2016) using Cas-Analyzer (Park *et al.*, 2017) and Cas-OFFinder (Bae *et al.*, 2014).

### Cell counting and chlorophyll determination

Cells in liquid media were counted with a Neubauer Bright Line hemocytometer and an Olympus CH30 microscope. The Chl content was determined spectrophotometrically in 100% (v/v) methanol extracts according to Holden (1976).

### Transmission electron microscopy

Cells were fixed with cold 5% glutaraldehyde buffered with 0.2 M sodium cacodylate at pH 6.8 for 1 h at 4 °C, and pre-embedded in 1% agar dissolved in distilled water. After solidification, they were post-fixed with sodium cacodylate buffer containing 1% OsO_4_ and 0.8% potassium ferricyanide for 1 h at 4 °C, and dehydrated at 4 °C using graded ethanol (from 50% to 100%). Specimens were then brought to room temperature and transferred through propylene oxide and Spurr’s embedding resin (Spurr, 1969) in propylene oxide. The specimens were moved to new pure resin and polymerized at 70 °C. Polymerized blocks were thin-sectioned using a PT-X ultramicrotome (RMC Boeckeler). Sections were collected on 0.25% (w/v) formvar-coated slot copper grids, stained with 3% (w/v) uranyl acetate and Reynold’s lead citrate (Reynolds, 1963), and examined and photographed using a JEM-1010 transmission electron microscope operated at 80 kV (JEOL). Images were recorded on Kodak EM Film 4489 and scanned to tagged image file format using an Epson Perfection V700 Photo scanner.

### Measurements of photosynthetic activity

Oxygen evolution was measured with a Clark-type oxygen electrode following the method described in Jeong *et al.* (2017) with the modification of illumination with incandescent light ranging from 25 to 1200 μmol photons m^−2^ s^−1^.

### Spectrophotometric and kinetic analysis

Thylakoid membranes were isolated as described in Kirst *et al.* (2012*a*). Spectrophotometric measurements of the amplitude of the light-minus-dark absorbance difference signal at 700 nm (P700) for PSI and 320 nm (Q_A_) for PSII were used to estimate the concentration of the photosystems in thylakoid membranes (Melis and Brown, 1980; Melis, 1989). Extinction coefficients at 700 nm (P700) were used as given by Hiyama and Ke (1972) and at 320 nm (Q_A_) as given by van Gorkom (1974). The kinetics of P700 photo-oxidation and Q_A_ photoreduction of 3-(3,4-dichlorophenyl)-1,1-dimethylurea (DCMU)-poisoned thylakoids were measured under weak but continuous green actinic excitation. First-order photoconversion kinetics were used to estimate the functional light-harvesting Chl antenna size of PSI and PSII (Melis and Thielen, 1980; Thielen and van Gorkom 1981; Melis, 1989, 1990, 1991). More specifically, the functional light-harvesting chlorophyll antenna size of *Chlamydomonas* PSI and PSII was measured from the first-order rate constants of P700 photo-oxidation and Q_A_ photoreduction, conducted upon weak continuous green actinic illumination of isolated thylakoid membranes (Polle *et al.*, 2000). For the functional PSII Chl antenna size, thylakoids were suspended in the presence of 10 μM DCMU, thereby blocking electron transport from Q_A_ to Q_B_ and the plastoquinone pool. For the functional PSI Chl antenna size, thylakoid membranes were suspended in the presence of DCMU, 200 μM potassium ferricyanide, and 100 μM methylviologen. The presence of ferricyanide ensured oxidation of the electron carriers between the two photosystems (e.g. the Rieske Fe–S center, cytochrome *f*, and plastocyanin), whereas methyl viologen acted as an efficient electron acceptor from the reducing side of PSI.

### SDS-PAGE and western blot analysis

SDS-PAGE analysis was carried out according to Laemmli (1970). Proteins were loaded on the basis of equal cell number. After protein separation, gels were stained with Coomassie Blue or blotted onto a polyvinylidene difluoride membrane (ATTO) in a semi-dry transfer system. Membranes were probed with antibodies against thylakoid membrane proteins. Immunodetection was performed using antibodies against LHCAs (Petroutsos *et al.*, 2011), LHCBs (Agrisera), PS core proteins (Agrisera), PetA (Agrisera), Atpβ (Agrisera), CrCpFTSY (Kirst *et al.*, 2012*a*), CrCpSRP43 (Kirst *et al.*, 2012*b*) and CrCpSRP54 (Jeong *et al.*, 2017). Polyclonal antibodies specific for CrLTD were generated in rabbit against two LTD peptides, CNFFKFGKNGFDSEAAGIVGS and GIVGSQGRDEYTYDDVEQYF (Abfrontier). Signals were visualized by using the WestSaveUp ECL Reagent (Abfrontier) and exposing the membranes to X-ray film. The National Institutes of Health ImageJ 1.48 software (https://imagej.nih.gov/ij/) was used for quantification of protein bands. To measure protein concentration, the DC Protein Assay kit (Bio-Rad) was used.

### Native Deriphat-PAGE and two-dimensional electrophoresis

Thylakoid membranes were solubilized at a Chl concentration of 0.5 mg ml^−1^ with *n*-dodecyl-α-D-maltoside (final concentration 1%), incubated on ice for 10 min and centrifuged at 20000 *g* for 10 min to remove unsolubilized material. Thylakoid membrane proteins (25 µg Chl per lane) were separated by gradient Deriphat-PAGE; the running gel had an acrylamide concentration gradient from 3.5 to 10.5% (w/v) (29:1 acrylamide–bisacrylamide) containing 12 mM Tris–HCl pH 8.5, 48 mM glycine, and a glycerol gradient from 10 to 14% (w/v). The stacking gel had 3.5% (w/v) acrylamide, 12 mM Tris–HCl pH 8.5, 48 mM glycine, and 10% (w/v) glycerol. The electrophoresis anode buffer was 12 mM Tris–HCl pH 8.3, 96 mM glycine. The cathode buffer had the same components as the anode buffer except for the addition of 0.1% (w/v) Deripat-160 (Cognis). The gel was electrophoresed at 50 V constant voltage overnight. For two-dimensional electrophoresis analysis, proteins were extracted from one-dimensional native Deriphat-PAGE strips by soaking them in SDS-PAGE stacking buffer containing 5 M urea twice for 25 min each and resolved by denaturing in a 12% SDS-polyacrylamide gel containing 2 M urea (second dimension). Acrylamide gels were stained with Coomassie Blue.

## Results

### Targeted *ltd* gene knockout using CRISPR–Cas9 technology

The *Chlamydomonas reinhardtii ltd* gene (Cre12.g551950) contains one exon, which is 504 bp in length, and encodes a protein of 167 amino acids including a 38-amino-acid- long chloroplast transit peptide predicted by Predalgo software (Tardif *et al.*, 2012). The CrLTD protein shares 54% identity and 70% similarity with its Arabidopsis homolog. It also contains an ankyrin domain (amino acids 105–137), already identified in Arabidopsis LTD (Cui *et al.*, 2011; Ouyang *et al.*, 2011). *Chlamydomonas reinhardtii* LTD knockout strains (*Crltd*) were generated by CRISPR–Cas9 methodology, comprising small insertions and deletions (indels) in this gene.

Four kinds of single guide RNAs (sgRNA) were designed using the Cas-Designer (Park *et al.*, 2015) to recognize and cleave the target gene (see Supplementary Table S1 at *JXB* online). Then, preassembled sgRNA and the Cas9 protein forming a CRISPR–Cas9 ribonucleoprotein (RNP) complex were transfected into the *C. reinhardtii* by electroporation. Because the Arabidopsis *ltd* knockout mutant has yellow leaves and lower Chl content than the wild-type (Ouyang *et al.*, 2011), *Chlamydomonas ltd* deletion mutants were initially selected on the basis of pale green coloration of transformant colonies (Supplementary Fig. S1). In a second screening step, the *ltd* gene knockout was confirmed by Sanger sequencing. Out of 388 colonies, four *ltd* knockout mutants were isolated, which was calculated to represent a 1.12% transformation efficiency.

**Fig. 1.**
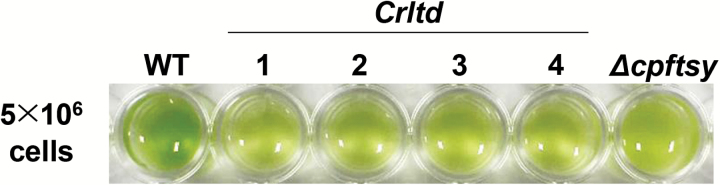
Coloration of *C. reinhardtii* wild-type (WT), *Crltd* strains 1–4, and the *Δcpftsy* strain. Cells were grown in TBP liquid media. At the same cell density (5 × 10^6^ cells ml^−1^), *Crltd* and *Δcpftsy* mutants showed a lighter green coloration, whereas the wild-type was dark green. The *Δcpftsy* strain, one of the light-harvesting antenna mutants generated by CRISPR–Cas9-mediated mutagenesis in our previous study (Baek *et al.*, 2016), was used as a control for comparison purposes.

**Fig. 2.**
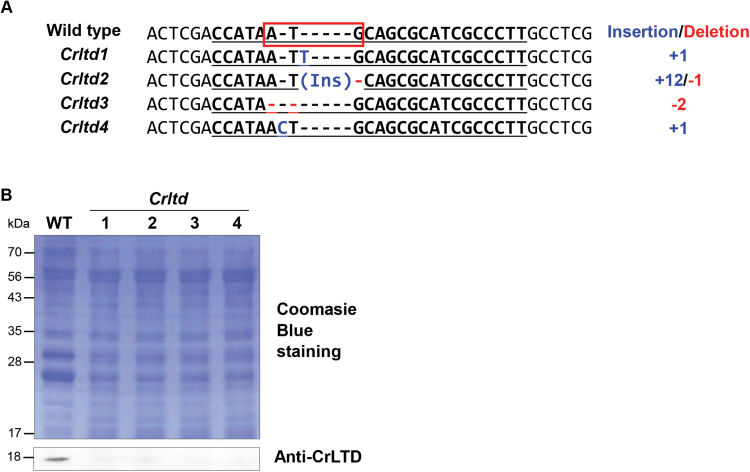
CRISPR–Cas9-mediated *ltd* gene disruption in *C. reinhardtii*. (A) Alignment of the DNA sequences of the wild-type and the *Crltd* mutants at the *ltd* locus. All mutants induced by CRISPR–Cas9 had small insertions and deletions (indels) in the *ltd* gene, which disrupted the start codon. The ATG start codon of the wild-type is indicated by the red box. (B) Coomassie-stained SDS-PAGE and western blot analysis with CrLTD-specific antibodies revealed that CRISPR–Cas9-induced mutations resulted in a deletion of the LTD protein in *Crltd* strains. Protein loading: 10 µg per lane. (This figure is available in color at *JXB* online.)

All *ltd* knockout mutants were isolated by using sgRNA 1 (see Supplementary Table S2). At the same cell density in liquid culture, the coloration of these mutants was lighter green compared with the wild-type and was similar to that of the *Δcpftsy* mutant (Baek *et al.*, 2016), which was used as a positive control in this study (Fig. 1). A variety of indel mutations were detected in the *ltd* locus of the four *ltd* knockout mutants (Fig. 2A; Supplementary Fig. S2), specifically so in the start codon of the *ltd* gene, which was targeted by the sgRNA 1. To test the expression of the LTD protein in the *ltd* stains, western blot analysis was performed with polyclonal antibodies raised against the LTD protein, the wild-type of which is a putative 18 kD protein (Fig. 2B). No protein–antibody cross-reaction signal could be detected in the 17–28 kDa region in any of the *ltd* stains. Therefore, all *ltd* strains showed the same phenotype in terms of lower pigmentation and lack of the LTD protein (Fig. 1). Absence of off-target mutations was examined by targeted deep sequencing (Supplementary Table S3). No indels were found at potential off-target sites that differed from on-target site by up to four nucleotides. A *Chlamydomonas ltd* mutant, designated as *Crltd1*, strain was selected for further investigation in this work.

### Cell growth analysis of the wild-type and *Crltd1* mutant

The rate of growth of the wild-type and *Crltd1* mutants was measured in photoautotrophic media under different light intensities (Table 1; Supplementary Fig. S3). At 100 μmol photons m^−2^ s^−1^ irradiance, *Crltd1* showed a growth rate of 1.37 ± 0.26 d^−1^, which was lower than that of wild-type (1.93 ± 0.03 d^−1^, Table 1). This retarded growth rate of the *Crltd1* strain is attributed to slower rates of light absorption, as compared with that of the wild-type (see below), consistent with the possibility that the *Crltd1* mutant possesses a truncated light harvesting antenna size. When the cells were exposed to a higher light intensity, i.e. 350 μmol photons m^−2^ s^−1^, the wild-type and *Crltd1* strains exhibited similar growth rates of 2.22 ± 0.07 and 2.03 ± 0.02 d^−1^, respectively, meaning that both strains harvested enough light energy to grow with similar rates. Furthermore, after 60 h of growth, the cell densities of the *Crltd1* mutant were 1.19-fold higher than that of the wild-type (Table 1).

**Table 1.** Growth characteristics of the wild-type and *Crltd1* mutant photoautotrophically grown at different light conditions

	100 μmol photons m^−2^ s^−1^		350 μmol photons m^−2^ s^−1^	
Parameter measured	Wild-type	*Crltd1*	Wild-type	*Crltd1*
Growth rate (μ d^−1^)	1.93 ± 0.03	1.37 ± 0.26	2.22 ± 0.07	2.03 ± 0.02
Cell density after 60 h of growth (10^7^ cells ml^−1^)	2.4 ± 0.37	1.39 ± 0.07	3.56 ± 0.22	4.25 ± 0.16

Values shown are means±SD (*n*=3).

### Chl content and composition in wild-type and the *Crltd1* mutant

**Fig. 3.**
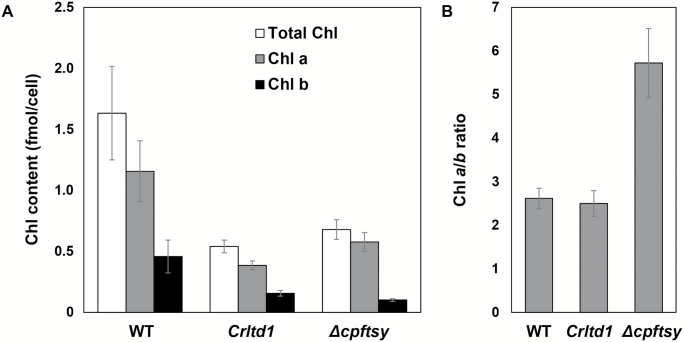
Total chlorophyll (Chl) content (A), and Chl *a*/*b* ratios (B) of the wild-type (WT) and the *Crltd1* and *Δcpftsy* strains (*n*≥3; values shown are means±SD). Note the lower Chl contents in *Crltd1* compared with the wild-type. The Chl *a*/*b* ratio of *Crltd1* was similar to that of the wild-type.

Chl content and composition of the wild-type, the *Δcpftsy* strain, and the *Crltd1* strain were measured in cultures grown photoautotrophically (Fig. 3). Results were essentially the same when cells were grown photoheterotrophically with supplemental organic carbon (results not shown). In the *Crltd1* strain, total Chl content per cell (0.54 fmol cell^−1^) was about 33% of that in the wild-type (1.63 fmol cell^−1^). However, the average Chl *a*/*b* ratio of the *Crltd1* strain (2.5 ± 0.3) was similar to that of the wild-type (2.62 ± 0.24). These pigmentation characteristics are unusual and unique for the *Crltd1* strain because the *Δcpftsy* mutant, as well as other Chl-deficient mutants examined so far (Kirst and Melis, 2014; Kirst *et al.*, 2017), exhibited lower Chl contents and a substantially elevated Chl *a*/*b* ratio in comparison with the wild-type (Fig. 3).

For example, the *Δcpftsy* mutant contained 0.58 fmol Chl *a* per cell (50% of the wild-type level) and 0.1 fmol Chl *b* per cell (22% of the wild-type level) (Fig. 3A) (see also Kirst *et al.*, 2012a;Baek *et al.*, 2016). The differential lowering of Chl *a* and *b* levels resulted in a much higher Chl *a*/*b* ratio in the *Δcpftsy* strain (Fig. 3B). In the *Crltd1* strain, the content of both Chl *a* and *b* was lowered proportionally, resulting in no discernable change in the Chl *a*/*b* ratio (Fig. 3B).

### Cell ultrastructure in wild-type and the *Crltd1* mutant

**Fig. 4.**
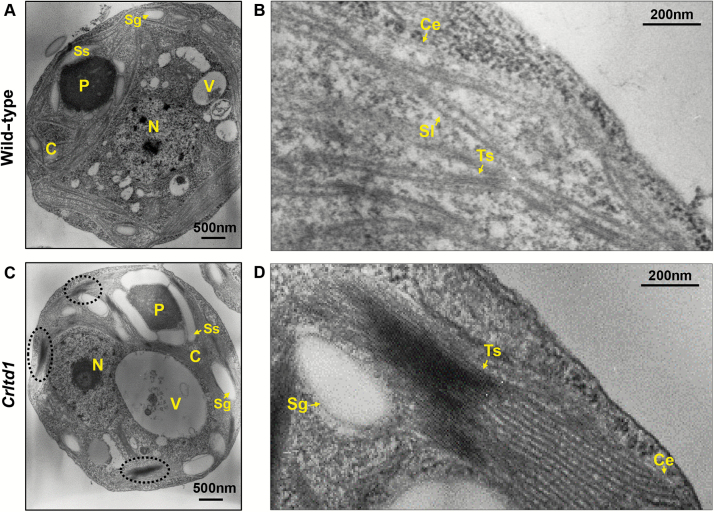
Transmission electron microscopy images of the cross-sections from wild-type (A, B) and *Crltd1* (C, D). N, nucleus; V, vacuole; C, chloroplast; Ce, chloroplast envelope; P, pyrenoid; Ss, starch sheath; Sg, starch granule; Ts, thylakoid stacks; Sl, stroma lamellae. *Crltd1* showed over-stacked thylakoid membranes (dotted circles in C) and an exceptionally large cytosolic vacuole. (This figure is available in color at *JXB* online.)

To investigate the cellular and subcellular properties of the *Chlamydomonas ltd* mutant, we performed transmission electron microscopy analysis. In both the wild-type and *Crltd1* grown photoheterotrophically, the chloroplast was observed to surround the nucleus and vacuole (Fig. 4A, C). Interestingly, in the *Crltd1* mutant, the vacuole was exceptionally large. The size of the vacuoles in seven to ten randomly selected cell images was 6 ± 2.53% of the total cell area in the wild-type and 10.45 ± 4.38% in the mutant cells. These values were significantly different from each-other (Student’s *t*-test, *P*<0.05). The wild-type thylakoid membranes formed stacks of two or more thylakoids and stroma lamellae composed of single thylakoids (Fig. 4B). On the other hand, extensive and dense grana-like thylakoid membrane layers were observed in the mutant with substantially smaller number of stroma-exposed lamellae (Fig. 4D).

### Photosynthetic activity of wild-type and *Crltd1* mutant

**Fig. 5.**
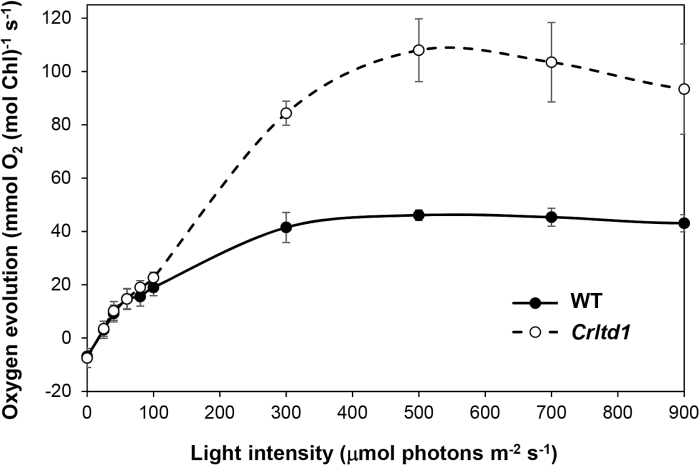
Light-saturation curves of photosynthesis in the wild-type (WT) and *Crltd1* (*n*≥3; values shown are means±SD). Measured on a per Chl basis, the rate of respiration of the *Crltd1* mutant was similar to that of the wild-type, but the light-saturated rate of oxygen evolution (*P*_max_) was about twice that of the wild-type. A 1 ml aliquot of cell suspension containing 2 μM Chl was loaded onto the oxygen electrode chamber.

Changes in photosynthetic pigment accumulation and structure of thylakoids may have affected the photosynthesis of the mutant. To investigate the function of the photosynthetic apparatus, we measured the light-saturation curves of photosynthesis by the oxygen evolution of cells grown photoautotrophically. In the dark, the rate of respiration of the *Crltd1* mutant was similar to that of the wild-type (Table 2). In the 20–100 μmol photons m^−2^ s^−1^ range of incident intensity, the mutant and the wild-type displayed similar rates of oxygen evolution per Chl per second (Fig. 5). However, on a per cell basis, the photosynthetic activity of the mutant was about 50% of that in the wild-type under the above light-limiting conditions. Therefore, under light-limiting conditions, absence of the *ltd* gene had no noticeable effect on the per Chl rate or quantum yield of photosynthesis but it negatively affected the photosynthetic capacity of the cells.

**Table 2.** Photosynthesis and respiration characteristics of wild-type and the *Crltd1* mutant grown photoautotrophically

Parameter	Wild-type	*Crltd*
Respiration (mmol oxygen (mol Chl)^−1^ s^−1^)	6.89 ± 2.14	7.48 ± 3.57
Respiration (fmol oxygen (10^6^ cells)^−1^ s^−1^)	3.66 ± 0.46	2.19 ± 1.10
*P*_max_ (mmol oxygen (mol Chl)^−1^ s^−1^)	47.8 ± 1.87	108.08 ± 11.97
*P*_max_ (fmol oxygen (10^6^ cell)^−1^ s^−1^)	33.22 ± 2.42	31.59 ± 2.34
Half-saturation intensity (mmol photons m^−2^ s^−1^)	289.01 ± 13.62	380.49 ± 20.54

Values shown are means±SD (*n*≥3).

The photosynthetic activity of the wild-type saturated at ~300 μmol photons m^−2^ s^−1^, whereas that of the mutant saturated at 500 μmol photons m^−2^ s^−1^ (Fig. 5). At saturating light intensities, the maximum photosynthetic capacity (*P*_max_) was 47.8 ± 1.87 mmol oxygen (mol Chl)^−1^ s^−1^ for the wild-type and 108.08 ± 11.97 mmol oxygen (mol Chl)^−1^ s^−1^ for the mutant. It was reported that mutant strains in which photosynthesis saturates at a higher light intensity could possess a smaller light-harvesting Chl antenna than the wild-type (Polle *et al.*, 2000, 2003; Kirst *et al.*, 2012a, b; Jeong *et al.*, 2017). We therefore expected that the *Crltd1* mutant also has a truncated Chl antenna and further characterized its photosystems and their Chl antenna size.

### Photosynthetic apparatus characterization of wild-type and *Crltd1* mutant

To measure the concentration of the photosystems and size of the light-harvesting antenna, we isolated thylakoid membranes from cultures grown photoautotrophically. The concentration of the photosystems was estimated spectrophotometrically by the Δ*A*_320nm_ light-minus-dark absorbance change for the electron acceptor Q_A_ of PSII and the Δ*A*_700nm_ light-minus-dark absorbance change for the PSI reaction center P700 (Melis and Brown, 1980). The kinetics of Q_A_ photoreduction and P700 photo-oxidation were also measured under weak green actinic light to determine the Chl antenna size of each photosystem (Melis, 1989).

The ratio of Q_A_ to total Chl was 2.13 ± 0.63 for the wild-type and 4.52 ± 0.72 (mmol:mol) in the *Crltd1* mutant, that is 212% greater than that of the wild-type. The ratio of P700 to total Chl was 1.65 ± 0.08 for the wild-type and 1.97 ± 0.34 (mmol:mol) in the mutant, that is 119% greater than that of the wild-type (Table 3). These phenotypes indicated that there is less Chl relative to the photosystems in this mutant, which may explain the higher light intensity needed to saturate the photosynthesis of the mutant.

**Table 3.** Photochemical apparatus characteristics of the wild-type and the *Crltd1* mutant grown photoautotrophically (TBP) or photoheterotrophically (TAP)

	TBP		TAP	
Parameter	Wild-type	*Crltd1*	Wild-type	*Crltd1*
Q_A_/total Chl (mmol:mol)	2.13 ± 0.63	4.52 ± 0.72	1.73 ± 0.25	3.43 ± 0.85
P700/total Chl (mmol:mol)	1.65 ± 0.08	1.97 ± 0.34	2.09 ± 0.31	2.29 ± 0.44
Q_A_/cell (mol (10^18^ cells)^−1^)	3.78 ± 1.23	2.52 ± 0.4	2.66 ± 0.39	1.86 ± 0.46
P700/cell (mol (10^18^ cells)^−1^)	3 ± 0.14	1.1 ± 0.19	3.21 ± 0.48	1.24 ± 0.24
PSII/PSI ratio	1.21	2.30	0.83	1.47
Functional PSII Chl antenna size	345.11 ± 16.22	191.96 ± 40.71	395.02 ± 22.83	249.02 ± 28.16
Functional PSI Chl antenna size	191.65 ± 13.67	77.43 ± 16.22	160.05 ± 11.57	83.46 ± 8.68

Values shown are means±SD (*n*≥4). The size of the Chl antennae of photosystems I and II and reaction center concentrations were measured spectrophotometrically.

Interesting quantitative tendencies in the abundance of PSI and PSII were found on a per cell basis. The content of Q_A_ per cell was lowered from 3.78 ± 1.23 (wild-type) to 2.52 ± 0.4 mol per 10^18^ cells in the *Crltd1* mutant, i.e. down to 67%. By contrast, the content of P700 per cell was lowered from 3 ± 0.14 (wild-type) to 1.1 ± 0.19 mol per 10^18^ cells in the mutant, i.e. down to 37%, revealing a disproportionate lowering in PSI content (Table 3). These mutation-induced changes altered the PSII/PSI ratio of the *Crltd1* mutant (2.3:1) relative to that of the wild-type (1.21:1) (Table 3). This alteration in the PSII/PSI ratio was noted in cells grown either autotrophically or heterotrophically on externally provided carbon source (Table 3). The results strengthen the notion that the ratio of the two photosystems depends in this case on the *ltd* antenna mutation but it does not depend on the carbon source during cell growth (Polle *et al.*, 2000). The functional light-harvesting Chl antenna size of PSII and PSI in the mutant were 55% and 40% of those in the wild-type, respectively. It was noted that, under all growth conditions, the Chl antenna size of PSI in the *Crltd1* mutant was essentially that of the PSI core complex (Glick and Melis, 1988), suggesting the total absence of the peripheral LHCA antenna from PSI in this mutant (Table 3).

### Western blot analysis of thylakoid membrane proteins in the *Crltd1* mutant

**Fig. 6.**
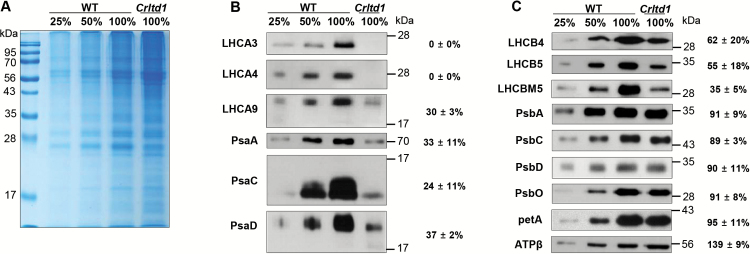
SDS-PAGE and western blot analysis of total thylakoid membrane proteins from the wild-type (WT) and *Crltd1* mutant. (A) A Coomassie-stained SDS-PAGE gel of total *C. reinhardtii* protein extracts. (B) Western blot analysis with specific polyclonal antibodies raised against PSI subunits of *C. reinhardtii*. (C) Western blot analysis with specific polyclonal antibodies raised against PSII, cytochrome *f* (PetA) and the β-subunit of ATP synthase (ATPβ) of *C. reinhardtii*. Relative amounts of proteins in the *Crltd1* strain compared with the wild-type are shown next to the protein bands (*n*≥3; values shown are means±SD). Loading of the gels: 8 × 10^5^ cells per lane (23.4 ± 2.6 µg of protein for WT and 23.3 ± 4 µg of protein for *Crltd1*). (This figure is available in color at *JXB* online.)

A lower photosystem content and diminished light-harvesting antenna size, especially those of PSI, in the *Crltd1* mutant would inevitably have resulted in altered amounts of thylakoid membrane proteins. We examined the amount of specific proteins by western blotting using cells grown photoautotrophically. Proteins were loaded on the basis of equal cell number and, for the wild-type, samples were loaded at three different concentrations (25, 50, and 100%=8 × 10^5^ cells per lane) to ensure linearity of the staining signal (Fig. 6A). The amount of each protein in the mutant relative to the wild-type was measured in at least three experiments, representative results of which are shown in Fig. 6B, C.

In the *Crltd1* mutant, no cross-reaction was observed with the LHCA3- and LHCA4-specific antibodies, and the abundance of LHCA9 was only 30 ± 3% or less of that in the wild-type (Fig. 6B). The amounts of LHCB4, LHCB5, and LHCBM5 in the mutant were 62 ± 20%, 55 ± 18%, and 35 ± 5%, respectively, of those measured in the wild-type (Fig. 6C). Overall, the content of LHCB proteins in the mutant was 50% of that in the wild-type, and the content of LHCA proteins was reduced even more severely to a very low level.

A dissimilar effect of the mutation was also noted on the photosystem content. The amounts of photosystem reaction center proteins in the mutant were lowered disproportionately for PSI than for PSII. In the mutant, levels of PSI core proteins PsaA, PsaC, and PsaD, which are associated with the LHCA, were 30% of those in the wild-type (Fig. 6B). Levels of the PSII core proteins PsbA, PsbC, PsbD, and PsbO were about 90% of those in the wild-type (Fig. 6C).

Qualitatively, the spectrophotometric Q_A_ per cell and P700 per cell measurements (Table 3) are in agreement with the western blot PsbA, PsbC, PsbD, and PsbO (for PSII) and PsaA, PsaC, and PsaD (for PSI) per cell data for wild-type and *ltd* mutant (Fig. 6). The small quantitative discrepancy between spectrophotometric and western blot results for PSII is probably due to the density of the bands in PsbA, PsbC, PsbD, and PsbO, which tends to minimize the difference between wild-type and *ltd* mutant.

To examine whether the cytochrome *b*_6_*f* complex was also affected in the mutant, western blot analyses with polyclonal antibodies against the cytochrome *f* protein (PetA) were conducted. Results showed no difference between wild-type and the *ltd* mutant in terms of Cyt *f* content (Fig. 6C). In contrast, the β-subunit of ATP synthase (ATPβ) was increased in the mutant to 139% of the level in the wild-type (Fig. 6C). This increment may be a consequence of the altered bioenergetic landscape in this strain. The levels of the CpSRP pathway proteins were similar in the wild-type and the *Crltd* mutant (see Supplementary Fig. S4).

### Analysis of photosystem complexes in wild-type and *Crltd1* mutant by Deriphat-PAGE and two-dimensional PAGE

**Fig. 7.**
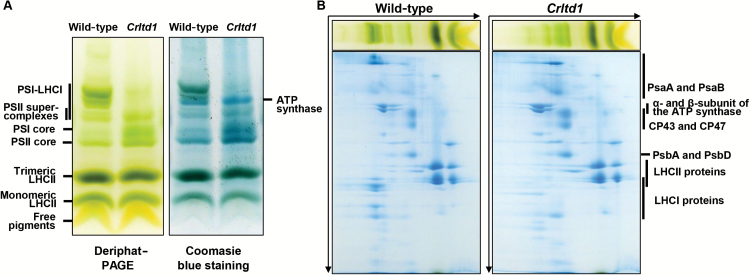
Analysis of thylakoid membrane protein complexes in the wild-type and *Crltd1* mutant by Deriphat-PAGE (A) and two-dimensional PAGE (B). Loading of the gels: 25 µg Chl per lane.

To gain further insight into the organization of photosynthetic complexes, we performed native Deriphat-PAGE analysis of the wild-type and *Crltd1* mutant. Thylakoid membranes of photoautotrophically grown cells were solubilized with 1% α-dodecyl maltoside and loaded on the basis of equal Chl. In the wild-type, we observed several green bands representing free pigments, monomeric LHCII, trimeric LHCII, PSII core, and PSI core (Fig. 7A, left panel), which resulted from sequential release of the LHC proteins from the photosystems (Järvi *et al.*, 2011). In addition, PSII supercomplexes and PSI–LHCI complexes of various sizes were observed in the slow electrophoretic mobility part of the gel. The PSI–LHCI complexes appeared to be larger and showed a broader range of electrophoretic mobility than the PSII supercomplexes.

In the *Crltd1* mutant, the PSI–LHCI complex was nearly absent compared with the wild-type, showing loss of PSI holocomplexes, consistent with the lower levels of PSI (Table 3) and LHCA (Fig. 6A) implying that loss of LHCI had a considerable effect on the formation of the PSI–LHCI complex. Instead, the abundance of the PSII supercomplexes, PSII core and ATP synthase complexes, which could be observed in a Coomassie-stained gel, was much higher than that in the wild-type (Fig. 7A, right panel).

Each native Deriphat-PAGE band was further characterized by western blotting with antibodies against PsaA, PsbA, LHCB, and LHCBM5 (see Supplementary Fig. S5) and two-dimensional PAGE analysis (Fig. 7B). Identification of the bands by western blotting after Deriphat-PAGE was consistent with the results of two-dimensional PAGE. For example, the presence of PSI–LHCI complexes in the mutant as faint bands on Deriphat gels agreed with a lower abundance of LHCI in two-dimensional PAGE. Keeping the α- and β-subunits of ATP synthase as migration references of the 2D-PAGE, it was clear that PsaA and PsaB in the wild-type were associated with a complex of a lower mobility than the ATP synthase in the first dimension. The opposite was instead observed with the *Crltd1* PsaA and PsaB, because of their association with a complex smaller than the ATP synthase. This *Crltd1* complex probably corresponds to a PSI without its peripheral antenna, because it contained the PSI core subunits but lacked the LHCI.

## Discussion

In the green lineage of eukaryotic photosynthetic organisms, the light-harvesting antennae of photosystems I and II consist of chloroplast-encoded core complexes and nuclear-encoded LHCPs peripheral to the core. LHCPs are imported from the cytosol, translocated across the chloroplast envelope, and then directed to the CpSRP pathway by the LTD protein. The latter has been identified as an ankyrin domain-containing protein in Arabidopsis (Cui *et al.*, 2011; Ouyang *et al.*, 2011). The CpSRP pathway facilitates the post-translational transport of these proteins to photosystem assembly sites in the thylakoid membrane (Henry, 2010). The function of the CpSRP pathway in green microalgae is analogous to that in higher plants, with several distinct and important differences (Kirst and Melis, 2014). It appears from this work that the function of the LTD protein in green microalgae is slightly different from its Arabidopsis homolog.

### Generation of null LTD from green algae

To elucidate the function of CrLTD and the effect of CrLTD deficiency on LHCP assembly *in vivo*, we generated *ltd* null mutants in *C. reinhardtii* by using CRISPR–Cas9 technology, which was recently successfully applied to *C. reinhardtii* (Baek *et al.*, 2016). This method has advantages over traditional mutagenesis approaches: generation of knockout or knockdown *C. reinhardtii* mutants by DNA insertional mutagenesis is time-consuming and labor-intensive (Polle *et al.*, 2003; Tetali *et al.*, 2007; Jinkerson and Jonikas, 2015; Baek *et al.*, 2016). RNA silencing approaches cannot completely suppress the expression of the target gene (Schroda, 2006). Generation of *Crltd* null mutants by transfecting a preassembled CRISPR–Cas9 complex is advantageous because this complex induces indel-type mutations at the target site, unlike random mutagenesis by vector insertion (Kim *et al.*, 2014). The transformation efficiency of the CRISPR–Cas9 method was calculated as 1.12% (see Supplementary Fig. S1). Furthermore, our sequencing analysis of the *Crltd* null mutant confirmed that the RNP complex cleaved only the *Crltd* locus but not any other part of the genome (Supplementary Table S3). Thus, the *Crltd* mutant generated by the CRISPR–Cas9 method allowed a clear observation of the specific effect(s) of the absence of the CrLTD protein because there were no other genetic lesions.

### Phenotype of the *C. reinhardtii* LTD mutant

The ankyrin domain is known to mediate protein–protein interactions (Mosavi *et al.*, 2004). In the CpSRP pathway, such interactions occur between the ankyrin domain of the CpSRP43 protein and the L18 motif of the LHCPs, or between the ankyrin domain of the LTD protein and the T14 motif of the LHCPs in Arabidopsis (DeLille *et al.*, 2000; Tu *et al.*, 2000; Stengel *et al.*, 2008; Cui *et al.*, 2011; Ouyang *et al.*, 2011). Therefore, it is suggested that the ankyrin domain of the LTD protein in *Chlamydomonas*, which has a high similarity to its Arabidopsis homolog, also participates in protein–protein interaction with the LHCPs in the chloroplast of this green microalga. Indeed, this work presented evidence that loss of LTD function quantitatively affected the LHCP assembly in the chloroplast of *Chlamydomonas*. This is shown in the results of Table 3, where the size of the functional PSI Chl antenna of the *Crltd1* mutant is strictly limited to that of the PSI reaction center core (Glick and Melis 1988) and the antenna totally lacks LHCI (see also Fig. 6B), in spite of the assembly of significant amounts of PSI.

The *Crltd1* mutant investigated in this work exhibited pale green coloration and had a lower total Chl content per cell than the wild-type (Figs 1 and 3). This phenotype is typical for null mutants lacking CpSRP pathway proteins both in Arabidopsis and *C. reinhardtii* and is caused by a lower level of LHCP assembly in the thylakoid membrane of photosynthesis (Amin *et al.*, 1999; Hutin *et al.*, 2002; Tzvetkova-Chevolleau *et al.*, 2007; Kirst *et al.*, 2012a, b; Jeong *et al.*, 2017). However, the *Crltd1* mutant displayed a profile of thylakoid membrane proteins (Fig. 6), which was different from the previously reported Arabidopsis *ltd* null mutant (Ouyang *et al.*, 2011). In the latter, levels of both LHCI and LHCII were considerably lower, and levels of the core PSI and PSII complexes were half of those in the wild-type control (Ouyang *et al.*, 2011). In the *Crltd1* mutant, LHCI was dramatically lowered to very low levels, whereas LHCII accumulated at 50% of the wild-type. In the *Crltd1* mutant, levels of the PSI core complex were 30% of that in the wild-type, but those of PSII were similar to wild-type. Consequently, the *Crltd1* mutant showed very low levels of PSI–LHCI holocomplex accumulation due to the very low LHCI content (Fig. 7). This phenotype resembles that of the Arabidopsis triple knockout mutant *ΔLhca*, in which four LHCA subunits were deleted (Bressan *et al.*, 2016), supporting the notion that *Crltd1* has a serious defect in LHCI and probably PSI core assembly.

The differential accumulation of PSI and PSII proteins in the *Crltd1* mutant is a unique and dissimilar feature in comparison to *Chlamydomonas* deletion mutants lacking CpSRP pathway proteins. A null mutant lacking CpFTSY, *Δcpftsy*, was also generated by the CRISPR–Cas9 method (Baek *et al.*, 2016), and LHCP content of both PSI and PSII was found to be lower than that in the wild-type (see Supplementary Fig. S4). Similarly, the *tla2* strain of *C. reinhardtii* (Kirst *et al.*, 2012*a*), in which the *CpFTSY* gene was disrupted by DNA insertional mutagenesis, possessed lower levels of both the LHCI and LHCII proteins (Kirst *et al.*, 2012*a*). The null mutants lacking CpSRP43 (*tla3*) and CpSRP54 (*tla4*) also showed uniformly lower levels of LHCI and LHCII (Kirst *et al.*, 2012*b*; Jeong *et al.*, 2017). However, *ac29-3*, a knockout mutant of the ALB3.1 translocase, exhibited a disproportionate lowering of LHCI compared with LHCII (Bellafiore *et al.*, 2002) and, in this respect, its phenotype was similar to the *Crltd1* mutant.

The unique profile of thylakoid membrane proteins in the *Crltd1* mutant is closely linked to its Chl composition. In all other CpSRP mutants examined to date, such as *tla2* (*Δcpftsy*), *tla3* (*ΔcpSRP43*), and *tla4* (*ΔcpSRP54*) (Kirst *et al.*, 2012a, b; Jeong *et al.*, 2017), the Chl *a*/*b* ratio was substantially elevated relative to that of the wild-type. Other Chl-deficient mutants affected in terms of their LHCP content or Chl biosynthesis also displayed a higher than wild-type Chl *a*/*b* ratio phenotype (Mussgnug *et al.*, 2007; Beckmann *et al.*, 2009; Bonente *et al.*, 2011; Perrine *et al.*, 2012). This was always attributed to the predominant loss of Chl *b* and the LHCB in these mutants. However, uniquely, the Chl *a*/*b* ratio of the *Crltd1* mutant was similar to that of the wild-type (Fig. 3). This is attributed to the near absence of LHCA and substantial lowering of the PSI core content in the thylakoid membrane of the *Crltd1* mutant. In general, the PSII core complex contains 37 Chl *a* molecules (Glick and Melis, 1988), and its peripheral light-harvesting antenna complex contains up to 200 Chl molecules, nearly equally divided between Chl *a* and Chl *b*. The PSI core complex contains 95 Chl *a* molecules (Glick and Melis, 1988) and its peripheral light-harvesting antenna complex contains an additional 100 Chl molecules, with the latter having a Chl *a*/*b* ratio of ~8:1 (Melis, 1990; Polle *et al.*, 2000). Selective loss of the LHCA and a substantially lower level of PSI core in the *Crltd1* mutant resulted in a proportionate lowering of Chl *a* and Chl *b* in this mutant and a pigment composition dominated by that of PSII, translating into a Chl *a*/*b* ratio similar to that of the wild-type.

### A pleotropic effect upon deletion of LTD in *C. reinhardtii*

A severe reduction in the level of the PSI holocomplex (PSI core and LHCI proteins) in the *Crltd1* mutant changed the overall structure of the chloroplast. Transmission electron microscopy analysis revealed substantial differences in thylakoid membrane ultrastructure between the wild-type and *Crltd1* mutant (Fig. 4). The number of appressed thylakoid membranes was increased in the mutant chloroplast. Considering the diminished grana stacking in the Arabidopsis *ltd* mutant, it is interesting that the thylakoid membrane structure of the *Crltd1* mutant changed in the opposite direction (Cui *et al.*, 2011; Ouyang *et al.*, 2011; Mitra *et al.*, 2012). The structure of thylakoid membranes and distribution of the photosystems are highly related. In general, PSII and PSI are predominantly located in the grana lamellae and stroma lamellae, respectively (Andersson and Anderson, 1980; Vallon *et al.*, 1986). Therefore, the predominance of the PSII complex in the *Crltd1* mutant corresponds with the preponderance of appressed (stacked) thylakoids, as they are more likely to contain PSII than PSI (Andersson and Anderson, 1980; Vallon *et al.*, 1986). In addition, exceptionally large vacuoles were observed in the cytosol of the *Crltd1* mutant, which may be a consequence of inhibition of LHCP import in the chloroplast and the need to sequester them in the vacuole prior to degradation (Fig. 4). In this respect, a knockdown strain of *C. reinhardtii* lacking the ALB3.2 translocase also showed enlarged vacuoles compared with the wild-type (Göhre *et al.*, 2006). In the latter, a lower level of ALB3.2 led to misfolding of thylakoid membrane proteins, triggering an extensive recycling of chloroplast proteins and necessitating their sequestration into vacuoles for degradation (Göhre *et al.*, 2006). In this process, LHCPs and other proteins, including the large subunit of Rubisco and the α-subunit of the ATP-synthase (both synthesized on plastid ribosomes), were also found in cytoplasmic vacuoles (Park *et al.*, 1999; Göhre *et al.*, 2006). It is possible that LHCP whose import has been inhibited or imported but unassembled stromal LHCPs are transported to the vacuoles by a general degradation pathway responsible for scavenging stromal proteins. Taken together, these observations suggest that the imbalance of chloroplast proteins caused by the severe down-regulation of PSI concentration and thylakoid membrane holocomplex assembly in the *Crltd1* mutant may induce this cytoplasmic vacuole enhancement. Thus, deletion of the *Crltd* protein appeared to have pleiotropic effects, emanating from the loss of a protein required for LHCP transport, the absence of which destabilized the entire photosynthetic complex assembly process and caused enhanced cytoplasmic vacuolation. However, the mechanism by which a lack of a single gene product elicits such far-reaching reorganization of cytosolic compartmentalization and thylakoid membrane restructuring in *C. reinhardtii* is not yet clear.

### The requirement for LTD may not be stringent in green microalgae

The *Crltd1* mutant exhibited photoautotrophic growth (Table 1; Supplementary Fig. S3), with a light-saturated rate of photosynthesis about the same as that of the wild-type (per cell basis, Table 2). This is at variance with the Arabidopsis *ltd* mutant, which was unable to grow photoautotrophically (Ouyang *et al.*, 2011).

Thus, despite the apparent similarity between the Arabidopsis LTD and *Chlamydomonas* LTD, phenotypes of the respective mutants are distinct and different from each other. The phenotype of the knockout mutant of the *LTD* gene in *C. reinhardtii* seemed to be leaky, suggesting flexible or redundant function of the CrLTD protein, as absence of this protein did not fully alleviate transport and assembly of PSI–LHCI complexes and photoautotrophic growth. Since light-harvesting antenna complexes not only harvest light energy for photosynthesis but also protect the photosynthetic apparatus from various environmental stresses (Erickson *et al.*, 2015; Wobbe *et al.*, 2016), especially in green algae, maintenance of the proper LHCP assembly may be required for survival in the wild. Arabidopsis and *C. reinhardtii* are under a substantially different developmental program. Arabidopsis chloroplasts stop developing when cell size reaches a maximum volume and chloroplast number increase stops (Jarvis and López-Jez, 2013). In *C. reinhardtii* possessing a single cup-shaped chloroplast, such developmental restrictions do not apply, as the cells are in a state of continuous growth and development. This fundamental developmental difference between the two species could account for the differences in LTD function between *C. reinhardtii* and Arabidopsis reported here.

Intriguingly, all mutations of individual CpSRP pathway components in *C. reinhardtii* caused mild to severe impairment of the pathway, affecting LHCP accumulation, but did not impair photoautotrophic growth (Bellafiore *et al.*, 2002; Ossenbühl *et al.*, 2004; Göhre *et al.*, 2006; Kirst *et al.*, 2012a, b; Jeong *et al.*, 2017). These phenotypes showed that most single-gene null mutations in the *Chlamydomonas* CpSRP pathway are leaky, unlike those in higher plants, in which the null mutations of CpFTSY and ALB3 proved to be lethal at the seedling stage (Asakura *et al.*, 2004; Asakura *et al.*, 2008). We cannot exclude the existence of an alternative pathway for LHCPs transport and assembly in *C. reinhardtii*, which may partially compensate for the loss of function of the CpSRPs. The presence of two *C. reinhardtii* ALB homologs (ALB3.1 and ALB3.2), unlike the single ALB in Arabidopsis, may support the possibility of an alternative pathway for LHCP assembly in *C. reinhardtii* (Bellafiore *et al.*, 2002; Göhre *et al.*, 2006). In this respect, generation of more than one mutant of the *Chlamydomonas* CpSRP pathway components may help to further dissect the mechanism(s) of LHCP transport and assembly in the thylakoid membrane of the green microalgae.

## Supplementary data

Supplementary data are available at *JXB* online.

Fig. S1. Visual examination of *C. reinhardtii* colonies in the course of screening for *Crltd* gene knockout mutant.

Fig. S2. Sanger sequencing chromatograms for CRISPR–Cas9-induced *Crltd* mutant strains.

Fig. S3. Growth curves of wild-type and the *Crltd1* mutant at different light intensities.

Fig. S4. Coomassie-stained SDS-PAGE of total proteins and western blot analysis of LHCPs and CpSRP components in the wild-type (WT) and *Crltd* strains.

Fig. S5. Analysis of thylakoid membrane protein complexes in the wild-type and *Crltd1* mutant.

Table S1. Target sequences of four sgRNAs used to recognize the *Crltd* gene.

Table S2. The mutation frequency (A) and pattern (B) of wild-type and RNP-transfected cells.

Table S3. Analysis of off-target effects in the wild-type and *Crltd1*.

Supplementary Tables and Figures

## Abbreviations:

Chl: chlorophyll
CpSRP: chloroplast signal recognition particle
Crltd: *C. reinhardtii ltd*
indel: insertions and deletion
LHCI: light-harvesting complex I
LHCII: light-harvesting complex II
LHCP: light-harvesting chlorophyll- and carotenoid-binding protein
LTD: LHCP translocation defect
RNP: ribonucleoprotein
sgRNA: single guide RNA
TAP: Tris–acetate–phosphate
TBP: Tris–bicarbonate–phosphate.
