# Supplementary material for: Deletion of the chloroplast LTD protein impedes LHCI import and PSI–LHCI assembly in Chlamydomonas reinhardtii
Source: J Exp Bot. 2017 Dec 30;69(5):1147–58. doi: 10.1093/jxb/erx457 (PMC6018721; doi:10.1093/jxb/erx457)

SUPPLEMENTARY MATERIAL

Deletion of the chloroplast LTD protein impedes LHCI import and PSI-LHCI assembly in *Chlamydomonas reinhardtii*

Jooyeon Jeong[1,6], Kwangryul Baek[1,6], Jihyeon Yu[2,6], Henning Kirst[3], Nico Betterle[3], Woongghi Shin[4], Sangsu Bae[5]*, Anastasios Melis[3]* and EonSeon Jin[1]*

[1] Department of Life Science and Research Institute for Natural Sciences, Hanyang University, Seoul, 04763, Korea

[2] School of Biological Sciences, Seoul National University, Seoul, 08826, Korea

[3] Department of Plant and Microbial Biology, University of California, Berkeley, California 94720-3102, USA

[4] Department of Biology, Chungnam National University, Daejeon, 34134, Korea

[5] Department of Chemistry, Hanyang University, Seoul, 04763, Korea

[6]These authors contributed equally to this article.

*Corresponding authors:

EonSeon Jin: Tel: +82-2-2220-2561; e-mail: esjin@hanyang.ac.kr

Anastasios Melis: Tel: +1-510-642-8166; e-mail: melis@berkeley.edu

Sangsu Bae: Tel: +82-2-2220-2618; e-mail: sangsubae@hanyang.ac.kr

Supplementary Table 1S. Target sequences of four sgRNAs used to recognize the *Crltd* gene.

	RGEN Target (5' to 3')	Position	Cleavage Position (%)	Direction	GC Contents (%, w/o PAM)	Out-of-frame Score	Mismatches				
							0	1	2	3	4
sgRNA1	AAGGGCGATGCGCTGCATTATGG	55	10.7	-	55	65.5	1	0	0	0	10
sgRNA2	ATGGCGCACAGTGCGAGCCACGG	97	18.1	-	65	68.9	1	0	0	0	14
sgRNA3	TCATCTTCTCGCCATATCTCGGG	132	26.3	+	45	60.3	1	0	0	0	1
sgRNA4	AAGTTGGCAGTCACATTGCGGGG	118	47.86	-	50	59.4	1	0	0	0	2

Supplementary Table 2S. The mutation frequency and pattern analysis of wild type and RNP-transfected cells. (A) The frequency of mutations (insertions and deletions; indels) in the wild-type (control) and RNP-transfected cells (experiment) measured for each sgRNA by targeted deep sequencing. (B) Mutation pattern analysis of RNP-transfected cells for sgRNA1. The top three indel patterns are listed. Patterns 1 and 3 were identical to those of *Crltd1* and *Crltd4*, respectively.

A.

	Target sequence	Sample	Total counts	Mutation counts	Mutation ratio (%)
sgRNA1	AAGGGCGATGCGCTGCATTATGG	control	55401	9	0.02%
		experiment	52027	913	1.75%
sgRNA2	ATGGCGCACAGTGCGAGCCACGG	control	55401	9	0.02%
		experiment	50897	9	0.02%
sgRNA3	TCATCTTCTCGCCATATCTCGGG	control	61508	2	0.00%
		experiment	53492	9	0.02%
sgRNA4	AAGTTGGCAGTCACATTGCGGGG	control	61508	2	0.00%
		experiment	46198	6	0.01%

B.

Top rank	Target sequence	Insertion/Deletion	Mutation counts
Wild type	ACTCGACCATAATGCAGCGCATCGCCCTTGCCTCG	-	-
1	ACTCGACCATAATTGCAGCGCATCGCCCTTGCCTCG	+1	222 counts
2	ACTCGACCATAAATGCAGCGCATCGCCCTTGCCTCG	+1	206 counts
3	ACTCGACCATAACTGCAGCGCATCGCCCTTGCCTCG	+1	123 counts

Supplementary Table 3S. Analysis of off-target effects in the wild type and *Crltd1*. Mutation frequencies at potential off-target sites of the *Crltd* gene-specific sgRNA1 were measured by targeted deep sequencing in the wild type and *Crltd1*. Potential off-target sites that differed from on-target sites by up to 4 nucleotides were selected. Different nucleotides between on-target and off-target are highlighted in Red.

**Target (5' to 3')**	**Wild type**			***Crltd1***		
	**Total reads**	**Indel reads**	**Indel frequency (%)**	**Total reads**	**Indel reads**	**Indel frequency (%)**
gAAGcCGATGCGCTaCgTTATGG	14321	0	0.0	8584	0	0.0
AAcGGCcAacCGCTGCATTACGG	27539	0	0.0	20148	0	0.0
AAGGGCGAgGCGCTGCtggAGGG	31882	2	0.006	21134	0	0.0
cAGGaCcATGCGCTGCAcTAGGG	37847	0	0.0	30822	0	0.0
AAGGGCGcTGtGCcGCAaTACGG	40377	0	0.0	27765	0	0.0
AAcGGCGcTGCGCTGCATcgTGG	29388	0	0.0	17057	0	0.0
AAcGGCGgcGCGCTGCAgTACGG	31603	0	0.0	10426	0	0.0

Supplementary Figure 1S. Visual examination of *C. reinhardtii* colonies in the course of screening for *Crltd* gene knockout mutant. Out of 388 colonies, 10 colonies were selected as the putative *ltd* knockout mutants (indicate with red circles). Among them, we finally identified four *ltd* knockout mutants by Sanger sequencing.

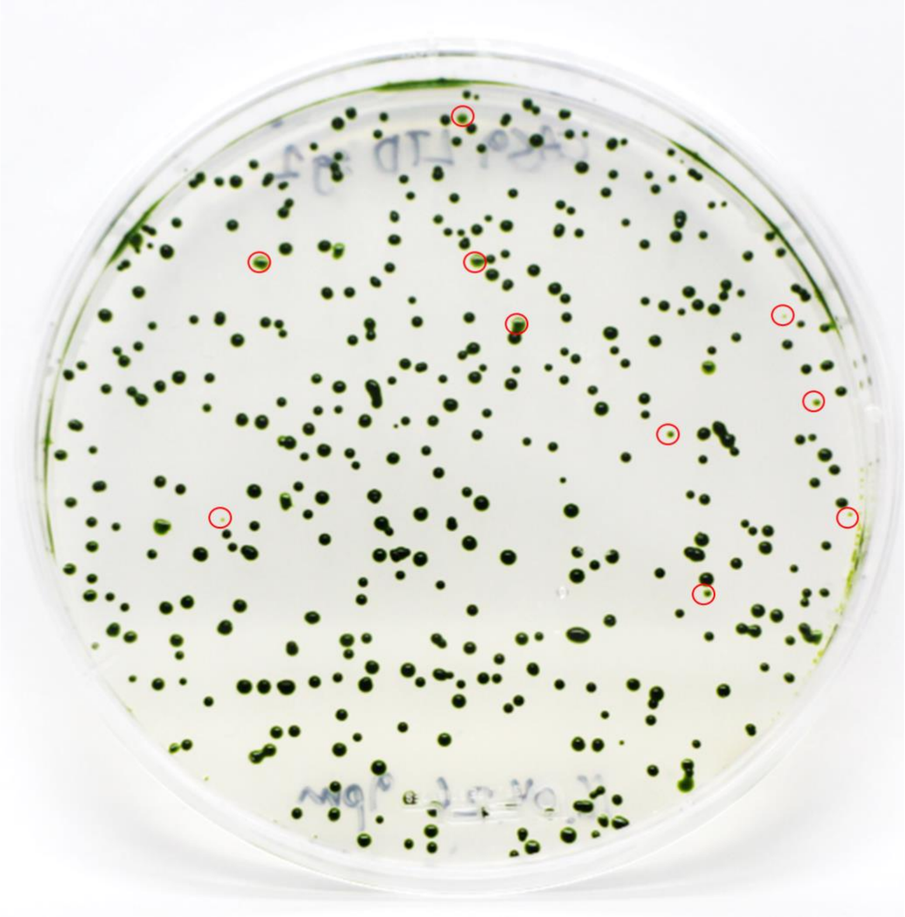

Supplementary Figure 2S. Sanger sequencing chromatograms for CRISPR-Cas9-induced *Crltd* mutant strains. *ltd* DNA sequences of four *Crltd* strains were confirmed by Sanger sequencing. The start codon of the wild type is indicated with a red box. Ins, insertions; del, deletions.

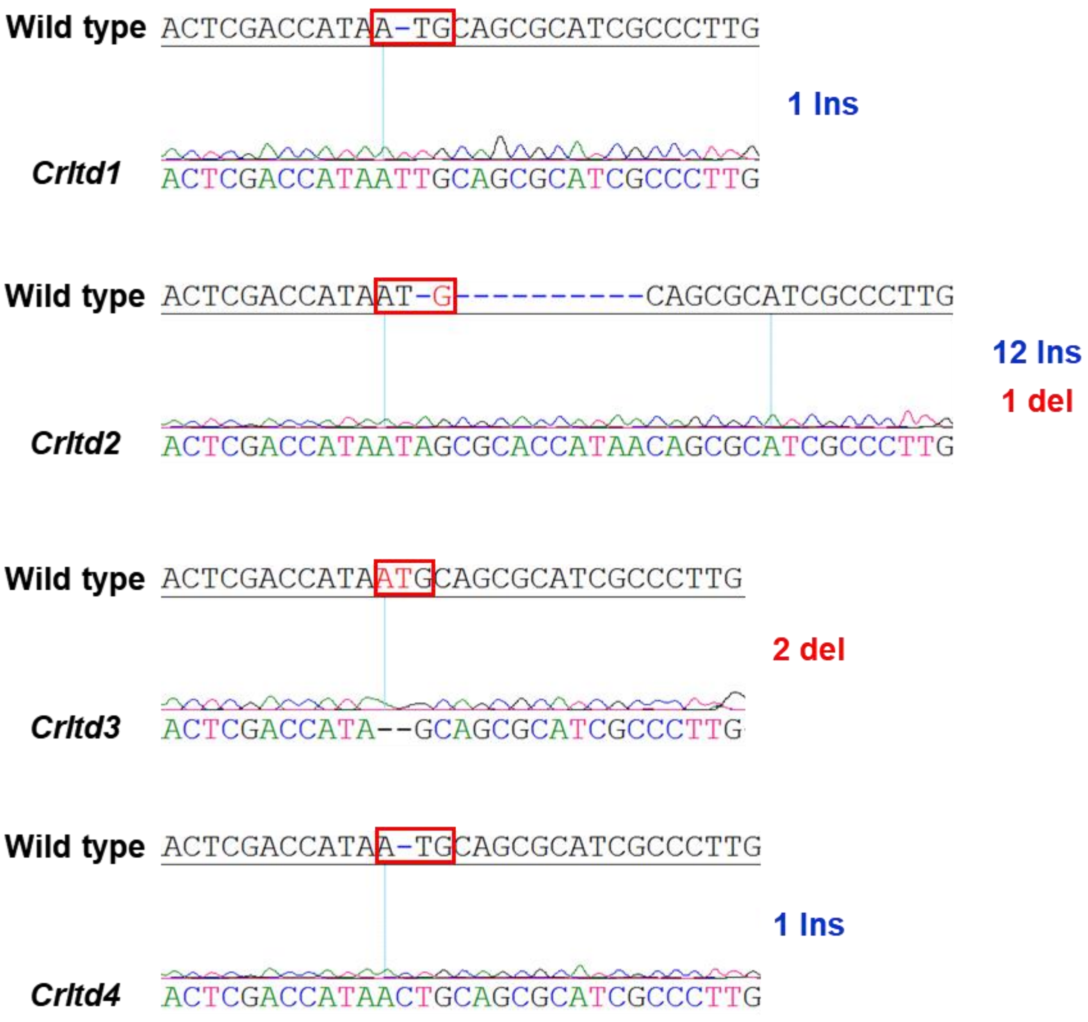

Supplementary Figure 3S. Growth curves of wild type (black) and *Crltd1* mutant (red) at different light intensities. Photoautotrophic growth under the light intensity of 100 µmol photons m^{-2} s^{-1} (A), 350 µmol photons m^{-2} s^{-1} (B) (n =3; values shown are means ± SD).

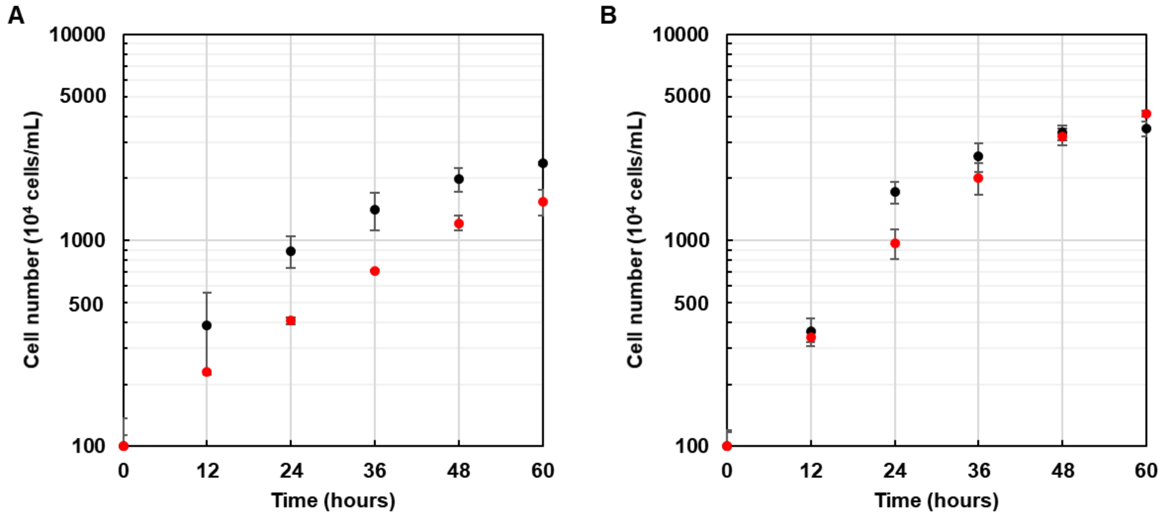

Supplementary Figure 4S. Coomassie-stained SDS-PAGE of total proteins and western blot analysis of LHCPs and CpSRP components in the wild type (WT) and *Crltd* strains. Cells were grown in TAP medium. Loading of the gel: 10^6 cells per lane.

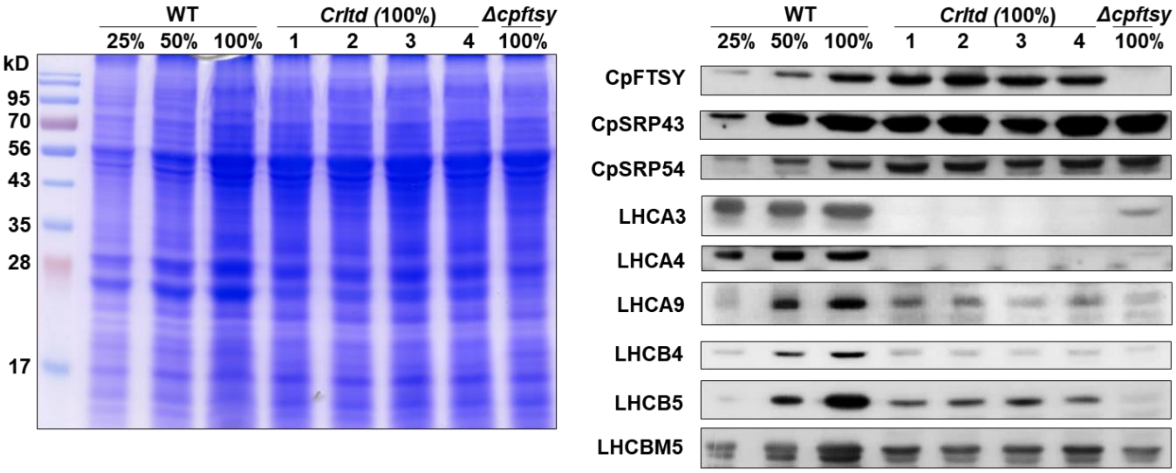

Supplementary Figure 5S. Analysis of thylakoid membrane protein complexes in the wild type and *Crltd1* mutant. The proteins were separated by Deriphat PAGE (1), Coomasie blue stained (2) and immunodetected with four antibodies, anti-PasA (3), anti-PsbA (4), anti-LHCB (5) and anti-LHCBM5 (6).

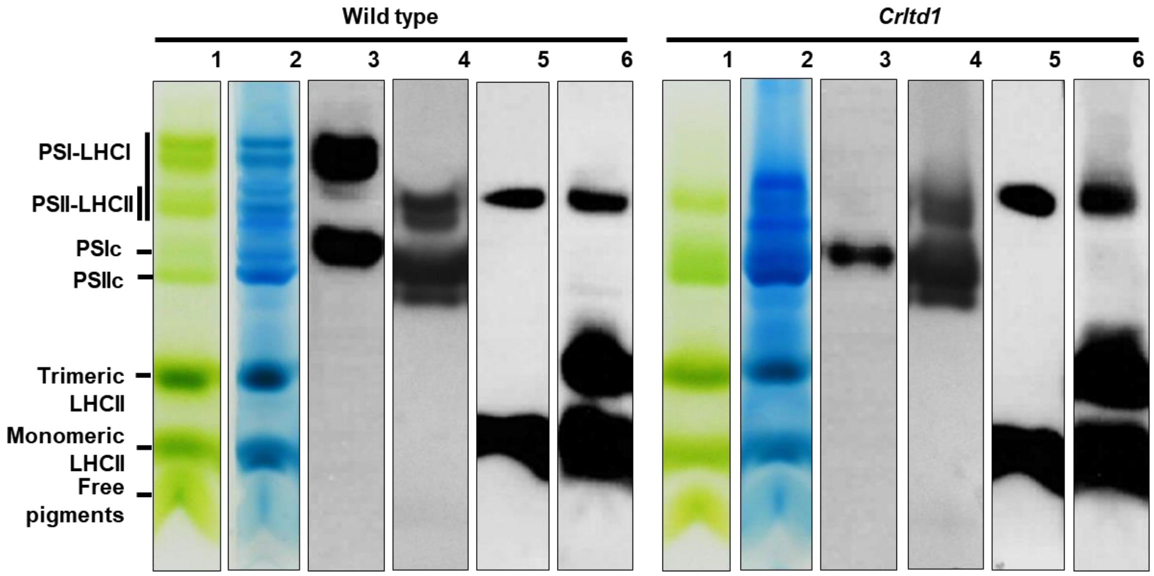

Supplement: Supplementary Tables and Figures [file erx457_suppl_supplementary_tables_and_figures.pdf]
